# Supplementary material for: Intestinal microbiome and maternal mental health: preventing parental stress and enhancing resilience in mothers
Source: Commun Biol. 2024 Feb 29;7:235. doi: 10.1038/s42003-024-05884-5 (PMC10904874; doi:10.1038/s42003-024-05884-5)
Supplement: Supplementary file 2 — Supplementary Information [file 42003_2024_5884_MOESM2_ESM.pdf]

Supplementary Materials

Supplementary Table 1. Comparison of the intestinal microbiota of healthy mothers and mothers with a high parenting stress risk (Study 1)

	All participants (N = 339)		Healthy Group (N = 274)		Parenting Stress Risk Group (N = 65)		Mann-Whitney *U* test (Stress risk vs Healthy)		
	Mean	*SD*	*Mean*	*SD*	*Mean*	*SD*	*W*	*p*-value	*q*-value
Shannon α	6.12	0.48	6.15	0.47	5.98	0.53	10482.00	0.026 *	0.019 *
Actinomyces	−4.02	0.82	−4.05	0.82	−3.90	0.81	7904.50	0.147	0.070 †
Bifidobacterium	−1.24	0.38	−1.24	0.31	−1.23	0.61	7617.00	0.070	0.040 †
Collinsella	−2.76	1.60	−2.70	1.57	−2.98	1.69	9313.00	0.559	0.114
Eggerthella	−2.95	0.78	−2.97	0.79	−2.87	0.77	8053.50	0.230	0.086 †
Bacteroides	−0.56	0.15	−0.56	0.16	−0.57	0.15	9742.00	0.239	0.086 †
Odoribacter	−3.14	1.16	−3.05	1.11	−3.50	1.31	10447.00	0.028 *	0.019 *
Alistipes	−2.22	1.20	−2.12	1.11	−2.63	1.45	10619.00	0.016 *	0.015 *
Parabacteroides	−1.88	1.08	−1.86	1.05	−1.95	1.24	8492.00	0.561	0.114
Erysipelatoclostridium	−2.81	0.79	−2.84	0.81	−2.66	0.72	7509.00	0.049 *	0.030 *
Turicibacter	−3.91	1.04	−3.9	1.03	−3.95	1.12	9187.00	0.679	0.128
Streptococcus	−2.09	0.68	−2.11	0.68	−2.03	0.72	8163.00	0.296	0.086 †
Intestinibacter	−3.44	1.04	−3.47	1.05	−3.32	1.01	8319.00	0.405	0.101
Romboutsia	−3.71	1.13	−3.71	1.12	−3.71	1.18	8683.00	0.746	0.134
Agathobacter	−3.07	1.55	−3.05	1.55	−3.14	1.54	9304.50	0.564	0.114
Anaerostipes	−2.06	0.74	−2.03	0.68	−2.20	0.93	9667.00	0.283	0.086
Blautia	−1.31	0.22	−1.31	0.22	−1.32	0.24	9201.00	0.677	0.128
Dorea	−2.38	1.03	−2.36	1.02	−2.46	1.11	9321.00	0.558	0.114
Fusicatenibacter	−2.44	1.29	−2.36	1.22	−2.81	1.53	9839.00	0.187	0.077 †
Lachnoclostridium	−1.86	0.36	−1.87	0.36	−1.81	0.37	8074.00	0.242	0.086 †
Lachnospira	−2.69	1.16	−2.63	1.14	−2.95	1.25	10419.00	0.033 *	0.021 *
Roseburia	−3.20	1.23	−3.16	1.22	−3.37	1.30	9816.00	0.195	0.077 †
Sellimonas	−3.01	1.12	−3.01	1.13	−3.01	1.12	8941.50	0.959	0.162
Tuzzerella	−3.89	1.05	−3.87	1.05	−3.98	1.06	9420.00	0.448	0.104
Lachnospiraceae_UCG-004	−3.59	1.20	−3.57	1.19	−3.69	1.22	9303.50	0.562	0.114
Colidextribacter	−3.05	1.06	−3.01	1.04	−3.21	1.12	9629.00	0.306	0.086 †
Flavonifractor	−2.96	0.75	−2.97	0.77	−2.90	0.67	8737.00	0.813	0.142
Oscillibacter	−2.89	0.92	−2.85	0.89	−3.03	1.03	9660.00	0.287	0.086 †
Butyricicoccus	−2.60	0.60	−2.56	0.55	−2.74	0.75	9880.50	0.170	0.075 †
Faecalibacterium	−1.53	0.98	−1.46	0.88	−1.79	1.29	10109.00	0.090	0.047 †
Negativibacillus	−3.94	1.04	−3.90	1.04	−4.12	1.04	9855.50	0.159	0.073 †
Subdoligranulum	−3.07	1.37	−3.03	1.34	−3.26	1.46	9495.00	0.401	0.101
UBA1819	−3.73	0.94	−3.70	0.92	−3.85	1.04	9686.00	0.265	0.086 †
Monoglobus	−2.91	1.15	−2.82	1.12	−3.28	1.21	11079.50	0.002 **	0.003 **
Phascolarctobacterium	−3.21	1.49	−3.11	1.46	−3.63	1.52	10533.50	0.018 *	0.015 *
Veillonella	−3.24	1.52	−3.32	1.50	−2.89	1.57	7287.00	0.019 *	0.015 *
Bilophila	−3.10	1.16	−3.05	1.13	−3.31	1.26	9672.00	0.276	0.086
Parasutterella	−3.18	1.46	−3.13	1.43	−3.40	1.61	9536.00	0.364	0.100
Sutterella	−3.04	1.64	−2.92	1.62	−3.53	1.64	10721.00	0.008 *	0.010 *
Escherichia-Shigella	−3.52	1.33	−3.60	1.32	−3.18	1.34	7306.00	0.020 *	0.015 *

**p or $q < 0.01$; *p or $q < 0.05$; †$q < 0.10$.

Supplementary Table 2. Comparison of the dietary habits of healthy mothers and mothers with a high parenting stress risk (Study 1)

	All participants (N = 339)		Healthy Group (N = 274)		Parenting Stress Risk Group (N = 65)		Mann-Whitney *U* test (Stress risk vs Healthy)		
	Mean	*SD*	*Mean*	*SD*	*Mean*	*SD*	*W*	*p*-value	*q*-value
staple food	4.52	0.90	4.53	0.91	4.48	0.89	9349.00	0.427	0.104
unrefined grain	1.40	0.94	1.42	0.96	1.32	0.85	9359.00	0.394	0.101
root vegetable	3.29	1.05	3.31	1.05	3.22	1.07	9379.50	0.488	0.110
green and yellow vegetables	3.06	1.05	3.04	1.03	3.17	1.13	8452.00	0.506	0.112
light colored vegetables	3.20	1.02	3.18	1.02	3.29	1.03	8387.50	0.449	0.104
fruits	2.63	1.07	2.64	1.06	2.62	1.10	9135.00	0.734	0.134
meat	3.50	0.97	3.54	0.95	3.32	1.06	9963.00	0.120	0.060 †
fish and shellfish	2.61	0.86	2.62	0.84	2.57	0.92	9471.50	0.391	0.101
egg	3.01	0.88	3.02	0.86	2.94	0.98	9237.00	0.622	0.123
milk and cheese	2.96	1.18	3.01	1.17	2.72	1.18	10104.00	0.078	0.043 †
yogurt and lactic fermenting beverage	2.27	1.06	2.28	1.06	2.23	1.07	9110.00	0.760	0.135
soy product	2.58	0.92	2.61	0.92	2.48	0.94	9592.50	0.298	0.086 †
natto	1.88	0.87	1.85	0.84	2.00	0.95	8231.50	0.307	0.086 †
pickled vegetables	1.70	0.76	1.72	0.74	1.65	0.82	9629.50	0.259	0.086 †
seaweed	2.10	0.77	2.08	0.73	2.18	0.92	8604.50	0.630	0.123
mushroom	2.58	0.87	2.57	0.86	2.62	0.93	8678.50	0.731	0.134
snack	3.45	1.01	3.47	1.01	3.37	1.02	9405.50	0.452	0.104
sugary drink	2.89	1.19	2.88	1.20	2.91	1.17	8794.00	0.872	0.150 †

†$q < 0.10$.

Supplementary Table 3. Comparing of the intestinal microbiota of healthy mothers and mothers with a high parenting stress risk with covariates (ANCOVA) (Study 1)

**Shannon α**	Sum Sq	df	*F*-value	*p*	
Group (PSI risk)	55321	1	5.56	0.02	*
mother's age	21299	1	2.14	0.14	
education	25245	1	2.54	0.11	
Residuals	3293224	331			

**Actinomyces**	Sum Sq	df	*F*-value	*p*	
Group (PSI risk)	22802	1	2.41	0.12	
mother's age	30478	1	3.23	0.07	.
education	419	1	0.04	0.83	
Residuals	3126007	331			

**Bifidobacterium**	Sum Sq	df	*F*-value	*p*	
Group (PSI risk)	25080	1	2.50	0.12	
mother's age	19004	1	1.89	0.17	
education	77	1	0.01	0.93	
Residuals	3326425	331			

**Collinsella**	Sum Sq	df	*F*-value	*p*	
Group (PSI risk)	4846	1	0.49	0.48	
mother's age	1957	1	0.20	0.66	
education	2386	1	0.24	0.62	
Residuals	3267802	331			

**Eggerthella**	Sum Sq	df	*F*-value	*p*	
Group (PSI risk)	15555	1	1.54	0.22	
mother's age	47905	1	4.73	0.03	*
education	6847	1	0.68	0.41	
Residuals	3352952	331			

**Bacteroides**	Sum Sq	df	*F*-value	*p*	
Group (PSI risk)	12247	1	1.22	0.27	
mother's age	3114	1	0.31	0.58	
education	8959	1	0.89	0.35	
Residuals	3325109	331			

**Odoribacter**	Sum Sq	df	*F*-value	*p*	
Group (PSI risk)	61664	1	6.43	0.01	*
mother's age	69525	1	7.25	0.01	**
education	2962	1	0.31	0.58	
Residuals	3175612	331			

**Alistipes**	Sum Sq	df	*F*-value	*p*	
Group (PSI risk)	68763	1	7.07	0.01	**
mother's age	37945	1	3.90	0.05	*
education	15195	1	1.56	0.21	
Residuals	3217184	331			

**Parabacteroides**	Sum Sq	df	*F*-value	*p*	
Group (PSI risk)	3673	1	0.36	0.55	
mother's age	35438	1	3.52	0.06	.
education	9	1	0.00	0.98	
Residuals	3333411	331			

**Erysipelatoclostridium**	Sum Sq	df	*F*-value	*p*	
Group (PSI risk)	38314	1	3.80	0.05	.
mother's age	455	1	0.05	0.83	
education	29	1	0.00	0.96	
Residuals	3335002	331			

**Turicibacter**	Sum Sq	df	*F*-value	*p*	
Group (PSI risk)	3885	1	0.42	0.52	
mother's age	12776	1	1.38	0.24	
education	30829	1	3.34	0.07	.
Residuals	3057971	331			

**Streptococcus**	Sum Sq	df	*F*-value	*p*	
Group (PSI risk)	5981	1	0.60	0.44	
mother's age	6065	1	0.61	0.44	
education	17551	1	1.75	0.19	
Residuals	3311713	331			

**Intestinibacter**	Sum Sq	df	*F*-value	*p*	
Group (PSI risk)	5729	1	0.58	0.45	
mother's age	25446	1	2.57	0.11	
education	15	1	0.00	0.97	
Residuals	3280878	331			

**Romboutsia**	Sum Sq	df	*F*-value	*p*	
Group (PSI risk)	1374	1	0.15	0.70	
mother's age	13386	1	1.43	0.23	
education	294	1	0.03	0.86	
Residuals	3104473	331			

**Agathobacter**	Sum Sq	df	*F*-value	*p*	
Group (PSI risk)	3158	1	0.33	0.57	
mother's age	17980	1	1.88	0.17	
education	14265	1	1.49	0.22	
Residuals	3172792	331			

**Anaerostipes**	Sum Sq	df	*F*-value	*p*	
Group (PSI risk)	15379	1	1.56	0.21	
mother's age	2632	1	0.27	0.61	
education	39933	1	4.04	0.05	*
Residuals	3268776	331			

**Blautia**	Sum Sq	df	*F*-value	*p*	
Group (PSI risk)	1285	1	0.13	0.72	
mother's age	1588	1	0.16	0.69	
education	14924	1	1.51	0.22	
Residuals	3278383	331			

**Dorea**	Sum Sq	df	*F*-value	*p*	
Group (PSI risk)	2937	1	0.29	0.59	
mother's age	18534	1	1.85	0.18	
education	5136	1	0.51	0.47	
Residuals	3320495	331			

**Fusicatenibacter**	Sum Sq	df	*F*-value	*p*	
Group (PSI risk)	21995	1	2.19	0.14	
mother's age	307	1	0.03	0.86	
education	3203	1	0.32	0.57	
Residuals	3326868	331			

**Lachnoclostridium**	Sum Sq	df	*F*-value	*p*	
Group (PSI risk)	14487	1	1.46	0.23	
mother's age	6529	1	0.66	0.42	
education	14028	1	1.41	0.24	
Residuals	3285198	331			

Lachnospira	Sum Sq	df	*F*-value	*p*	
Group (PSI risk)	50084	1	5.03	0.03	*
mother's age	13889	1	1.40	0.24	
education	36	1	0.00	0.95	
Residuals	3295561	331			

Roseburia	Sum Sq	df	*F*-value	*p*	
Group (PSI risk)	15187	1	1.53	0.22	
mother's age	13212	1	1.33	0.25	
education	3327	1	0.33	0.56	
Residuals	3291236	331			

Sellimonas	Sum Sq	df	*F*-value	*p*	
Group (PSI risk)	2	1	0.00	0.99	
mother's age	40589	1	4.02	0.05	*
education	124	1	0.01	0.91	
Residuals	3339067	331			

Tuzzerella	Sum Sq	df	*F*-value	*p*	
Group (PSI risk)	7418	1	0.81	0.37	
mother's age	38325	1	4.20	0.04	*
education	10442	1	1.14	0.29	
Residuals	3021255	331			

Lachnospiraceae_UCG-004	Sum Sq	df	*F*-value	*p*	
Group (PSI risk)	1916	1	0.20	0.65	
mother's age	10529	1	1.13	0.29	
education	7481	1	0.80	0.37	
Residuals	3096129	331			

Colidextribacter	Sum Sq	df	*F*-value	*p*	
Group (PSI risk)	13492	1	1.36	0.24	
mother's age	41820	1	4.22	0.04	*
education	20118	1	2.03	0.16	
Residuals	3282786	331			

Flavonifractor	Sum Sq	df	*F*-value	*p*	
Group (PSI risk)	310	1	0.03	0.86	
mother's age	42133	1	4.20	0.04	*
education	74	1	0.01	0.93	
Residuals	3319182	331			

Oscillibacter	Sum Sq	df	*F*-value	*p*	
Group (PSI risk)	12735	1	1.32	0.25	
mother's age	115664	1	11.97	0.00	***
education	66165	1	6.85	0.01	**
Residuals	3199324	331			

Butyricicoccus	Sum Sq	df	*F*-value	*p*	
Group (PSI risk)	12989	1	1.30	0.25	
mother's age	22195	1	2.23	0.14	
education	784	1	0.08	0.78	
Residuals	3297273	331			

Faecalibacterium	Sum Sq	df	*F*-value	*p*	
Group (PSI risk)	29490	1	2.94	0.09	.
mother's age	4900	1	0.49	0.49	
education	5754	1	0.57	0.45	
Residuals	3323852	331			

Negativibacillus	Sum Sq	df	*F*-value	*p*	
Group (PSI risk)	18200	1	1.98	0.16	
mother's age	1527	1	0.17	0.68	
education	2274	1	0.25	0.62	
Residuals	3038863	331			

Subdoligranulum	Sum Sq	df	*F*-value	*p*	
Group (PSI risk)	10782	1	1.11	0.29	
mother's age	1851	1	0.19	0.66	
education	1433	1	0.15	0.70	
Residuals	3216501	331			

UBA1819	Sum Sq	df	*F*-value	*p*	
Group (PSI risk)	13351	1	1.42	0.24	
mother's age	122603	1	13.00	0.00	***
education	32637	1	3.46	0.06	.
Residuals	3122195	331			

Monoglobus	Sum Sq	df	*F*-value	*p*	
Group (PSI risk)	96602	1	9.77	0.00	**
mother's age	7272	1	0.74	0.39	
education	2448	1	0.25	0.62	
Residuals	3273019	331			

Phascolarctobacterium	Sum Sq	df	*F*-value	*p*	
Group (PSI risk)	50195	1	5.27	0.02	*
mother's age	1653	1	0.17	0.68	
education	7741	1	0.81	0.37	
Residuals	3151857	331			

Veillonella	Sum Sq	df	*F*-value	*p*	
Group (PSI risk)	53372	1	5.69	0.02	*
mother's age	5612	1	0.60	0.44	
education	7517	1	0.80	0.37	
Residuals	3103940	331			

Bilophila	Sum Sq	df	*F*-value	*p*	
Group (PSI risk)	13637	1	1.39	0.24	
mother's age	23986	1	2.45	0.12	
education	11703	1	1.20	0.27	
Residuals	3236283	331			

Parasutterella	Sum Sq	df	*F*-value	*p*	
Group (PSI risk)	7940	1	0.83	0.36	
mother's age	6349	1	0.66	0.42	
education	10320	1	1.07	0.30	
Residuals	3178658	331			

Sutterella	Sum Sq	df	*F*-value	*p*	
Group (PSI risk)	62343	1	6.64	0.01	*
mother's age	2001	1	0.21	0.64	
education	7268	1	0.77	0.38	
Residuals	3107737	331			

Escherichia-Shigella	Sum Sq	df	*F*-value	*p*	
Group (PSI risk)	52642	1	5.67	0.02	*
mother's age	3801	1	0.41	0.52	
education	4136	1	0.45	0.50	
Residuals	3072628	331			

***p <0.001, **p <0.01; *p <0.05.

Supplementary Table 4. The prevalent microbiome found among Japanese mothers of children aged 0–4 years (Study 1)

Prevalent microbiota_genus	Kingdom	Phylum	Class	Order	Family	Genus
Actinomyces	Bacteria	Actinobacteria	Actinobacteria	Actinomycetales	Actinomycetaceae	Actinomyces
Agathobacter	Bacteria	Firmicutes	Clostridia	Clostridiales	Lachnospiraceae	Agathobacter
Alistipes	Bacteria	Bacteroidetes	Bacteroidia	Bacteroidales	Rikenellaceae	Alistipes
Anaerostipes	Bacteria	Firmicutes	Clostridia	Clostridiales	Lachnospiraceae	Anaerostipes
Bacteroides	Bacteria	Bacteroidetes	Bacteroidia	Bacteroidales	Bacteroidaceae	Bacteroides
Bifidobacterium	Bacteria	Actinobacteria	Actinobacteria	Bifidobacteriales	Bifidobacteriaceae	Bifidobacterium
Bilophila	Bacteria	Proteobacteria	Deltaproteobacteria	Desulfovibrionales	Desulfovibrionaceae	Bilophila
Blautia	Bacteria	Firmicutes	Clostridia	Clostridiales	Lachnospiraceae	Blautia
Butyricicoccus	Bacteria	Firmicutes	Clostridia	Clostridiales	Ruminococcaceae	Butyricicoccus
Colidextribacter	Bacteria	Firmicutes	Clostridia	Clostridiales	Oscillospiraceae	Colidextribacter
Collinsella	Bacteria	Actinobacteria	Coriobacteriia	Coriobacteriales	Coriobacteriaceae	Collinsella
Dorea	Bacteria	Firmicutes	Clostridia	Clostridiales	Lachnospiraceae	Dorea
Eggerthella	Bacteria	Actinobacteria	Coriobacteriia	Coriobacteriales	Coriobacteriaceae	Eggerthella
Erysipelatoclostridium	Bacteria	Firmicutes	Bacilli	Erysipelotrichales	Erysipelatoclostridiaceae	Erysipelatoclostridium
Escherichia-Shigella	Bacteria	Proteobacteria	Gammaproteobacteria	Enterobacteriales	Enterobacteriaceae	Escherichia-Shigella
Faecalibacterium	Bacteria	Firmicutes	Clostridia	Clostridiales	Ruminococcaceae	Faecalibacterium
Flavonifractor	Bacteria	Firmicutes	Clostridia	Clostridiales	Oscillospiraceae	Flavonifractor
Fusicatenibacter	Bacteria	Firmicutes	Clostridia	Clostridiales	Lachnospiraceae	Fusicatenibacter
Intestinibacter	Bacteria	Firmicutes	Clostridia	Clostridiales	Clostridiaceae	Intestinibacter
Lachnoclostridium	Bacteria	Firmicutes	Clostridia	Clostridiales	Lachnospiraceae	Lachnoclostridium
Lachnospira	Bacteria	Firmicutes	Clostridia	Clostridiales	Lachnospiraceae	Lachnospira
Lachnospiraceae_UCG-004	Bacteria	Firmicutes	Clostridia	Clostridiales	Lachnospiraceae	UCG-004
Monoglobus	Bacteria	Firmicutes	Clostridia	Monoglobales	Monoglobaceae	Monoglobus
Negativibacillus	Bacteria	Firmicutes	Clostridia	Clostridiales	Ruminococcaceae	Negativibacillus
Odoribacter	Bacteria	Bacteroidetes	Bacteroidia	Bacteroidales	Marinifilaceae	Odoribacter
Oscillibacter	Bacteria	Firmicutes	Clostridia	Clostridiales	Oscillospiraceae	Oscillibacter
Parabacteroides	Bacteria	Bacteroidetes	Bacteroidia	Bacteroidales	Tannerellaceae	Parabacteroides
Parasutterella	Bacteria	Proteobacteria	Gammaproteobacteria	Burkholderiales	Sutterellaceae	Parasutterella
Phascolarctobacterium	Bacteria	Firmicutes	Negativicutes	Acidaminococcales	Acidaminococcaceae	Phascolarctobacterium
Romboutsia	Bacteria	Firmicutes	Clostridia	Clostridiales	Clostridiaceae	Romboutsia
Roseburia	Bacteria	Firmicutes	Clostridia	Clostridiales	Lachnospiraceae	Roseburia
Sellimonas	Bacteria	Firmicutes	Clostridia	Clostridiales	Lachnospiraceae	Sellimonas
Streptococcus	Bacteria	Firmicutes	Bacilli	Lactobacillales	Streptococcaceae	Streptococcus
Subdoligranulum	Bacteria	Firmicutes	Clostridia	Clostridiales	Ruminococcaceae	Subdoligranulum
Sutterella	Bacteria	Proteobacteria	Gammaproteobacteria	Burkholderiales	Sutterellaceae	Sutterella
Turicibacter	Bacteria	Firmicutes	Bacilli	Erysipelotrichales	Erysipelotrichaceae	Turicibacter
Tuzzerella	Bacteria	Firmicutes	Clostridia	Clostridiales	Lachnospiraceae	Tuzzerella
UBA1819	Bacteria	Firmicutes	Clostridia	Clostridiales	Ruminococcaceae	UBA1819
Veillonella	Bacteria	Firmicutes	Negativicutes	Veillonellales	Veillonellaceae	Veillonella

Supplementary Table 5. The prevalent microbiome found among Japanese primiparous mothers in the early postpartum period (within 3–6 months) (Study 2)

Prevalent microbiota_genus	Kingdom	Phylum	Class	Order	Family	Genus
Actinomyces	Bacteria	Actinobacteria	Actinobacteria	Actinomycetales	Actinomycetaceae	Actinomyces
[Eubacterium]	Bacteria	Firmicutes	Erysipelotrichi	Erysipelotrichales	Erysipelotrichaceae	[Eubacterium]
Adlercreutzia	Bacteria	Actinobacteria	Coriobacteriia	Coriobacteriales	Coriobacteriaceae	Adlercreutzia
Clostridiaceae_Clostridium	Bacteria	Firmicutes	Clostridia	Clostridiales	Clostridiaceae	Clostridium
Lachnospiraceae_Clostridium	Bacteria	Firmicutes	Clostridia	Clostridiales	Lachnospiraceae	Clostridium
Ruminococcus	Bacteria	Firmicutes	Clostridia	Clostridiales	Ruminococcaceae	Ruminococcus
Erysipelotrichaceae_Clostridium	Bacteria	Firmicutes	Erysipelotrichi	Erysipelotrichales	Erysipelotrichaceae	Clostridium
Bacteroides	Bacteria	Bacteroidetes	Bacteroidia	Bacteroidales	Bacteroidaceae	Bacteroides
Barnesiella	Bacteria	Bacteroidetes	Bacteroidia	Bacteroidales	[Barnesiellaceae]	Barnesiella
Bifidobacterium	Bacteria	Actinobacteria	Actinobacteria	Bifidobacteriales	Bifidobacteriaceae	Bifidobacterium
Bilophila	Bacteria	Proteobacteria	Deltaproteobacteria	Desulfovibrionales	Desulfovibrionaceae	Bilophila
Blautia	Bacteria	Firmicutes	Clostridia	Clostridiales	Lachnospiraceae	Blautia
Butyricimonas	Bacteria	Bacteroidetes	Bacteroidia	Bacteroidales	[Odoribacteraceae]	Butyricimonas
cc_115	Bacteria	Firmicutes	Erysipelotrichi	Erysipelotrichales	Erysipelotrichaceae	cc_115
Collinsella	Bacteria	Actinobacteria	Coriobacteriia	Coriobacteriales	Coriobacteriaceae	Collinsella
Coprobacillus	Bacteria	Firmicutes	Erysipelotrichi	Erysipelotrichales	Erysipelotrichaceae	Coprobacillus
Coprococcus	Bacteria	Firmicutes	Clostridia	Clostridiales	Lachnospiraceae	Coprococcus
Eggerthella	Bacteria	Actinobacteria	Coriobacteriia	Coriobacteriales	Coriobacteriaceae	Eggerthella
Faecalibacterium	Bacteria	Firmicutes	Clostridia	Clostridiales	Ruminococcaceae	Faecalibacterium
Holdemania	Bacteria	Firmicutes	Erysipelotrichi	Erysipelotrichales	Erysipelotrichaceae	Holdemania
Lactonifactor	Bacteria	Firmicutes	Clostridia	Clostridiales	Lachnospiraceae	Lactonifactor
Odoribacter	Bacteria	Bacteroidetes	Bacteroidia	Bacteroidales	[Odoribacteraceae]	Odoribacter
Oscillospira	Bacteria	Firmicutes	Clostridia	Clostridiales	Ruminococcaceae	Oscillospira
Parabacteroides	Bacteria	Bacteroidetes	Bacteroidia	Bacteroidales	Porphyromonadaceae	Parabacteroides
Phascolarctobacterium	Bacteria	Firmicutes	Clostridia	Clostridiales	Veillonellaceae	Phascolarctobacterium
Streptococcus	Bacteria	Firmicutes	Bacilli	Lactobacillales	Streptococcaceae	Streptococcus
Sutterella	Bacteria	Proteobacteria	Betaproteobacteria	Burkholderiales	Alcaligenaceae	Sutterella
Turicibacter	Bacteria	Firmicutes	Bacilli	Turicibacterales	Turicibacteraceae	Turicibacter

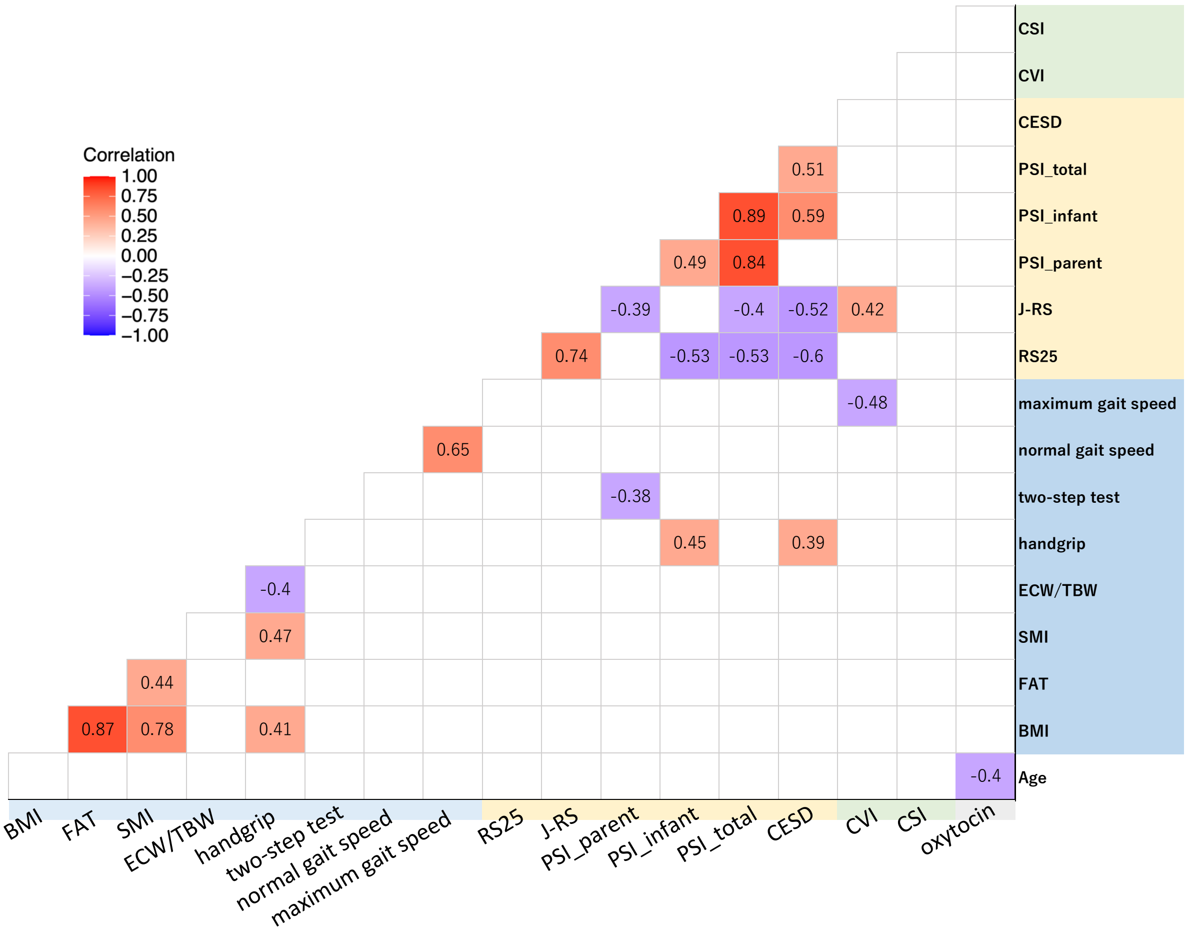

Supplementary Figure 1. Pearson's correlations between the physical functions, physiological indices, and psychological indices of postpartum mothers (Study 2)

Values in the figure are *r* with $p < 0.05$.

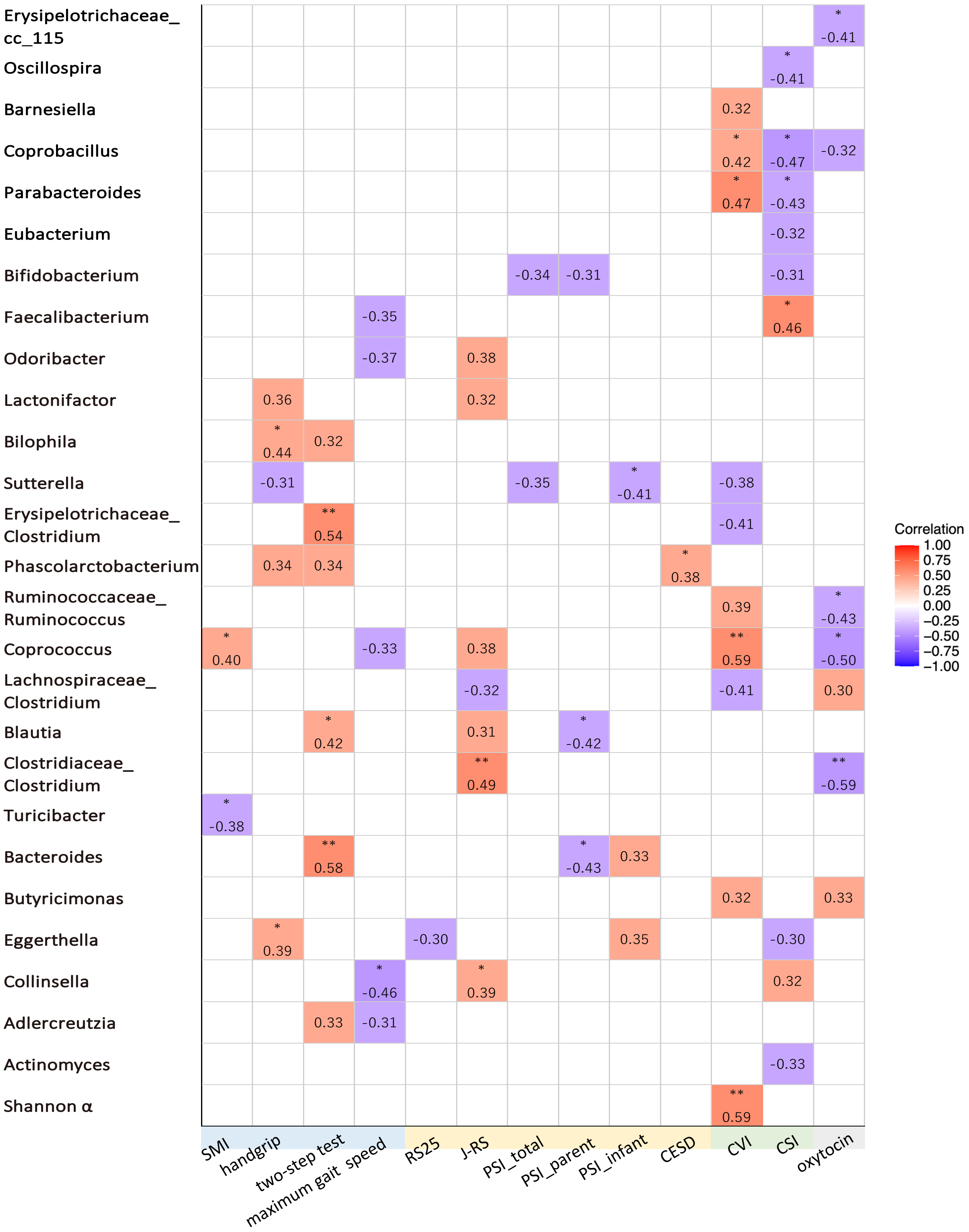

Supplementary Figure 2. Pearson's correlations between the intestinal microbiota, physical indices, and psychological indices of mothers (Study 2)

Values in the figure are all *r* with either a significant *p*-value or a medium to large effect size.

***p* <0.01; **p* <0.05.

	Subject																		
**Clostridium**	**s1**	**s2**	**s3**	**s4**	**s5**	**s6**	**s7**	**s8**	**s9**	**s10**	**s11**	**s12**	**s13**	**s14**	**s15**	**s16**	**s17**	**s18**	**s19**
**Clostridium sp. SY8519**	**0.00082**	**0.00029**	**0.00069**	**0.00106**	**0.00099**	**0.00038**	**0.00095**	**0.00060**	**0.00020**	**0.00049**	**0.00056**	**0.00066**	**0.00019**	**0.00089**	**0.00036**	**0.00014**	**0.00069**	**0.00016**	**0.00087**
Clostridium aceticum	0.00003	0.00001	0.00002	0.00005	0.00002	0.00002	0.00003	0.00003	0.00001	0.00002	0.00001	0.00003	0.00002	0.00005	0.00002	0.00001	0.00003	0.00001	0.00003
Clostridium acetobutylicum	0.00002	0.00001	0.00002	0.00002	0.00002	0.00001	0.00001	0.00003	0.00001	0.00001	0.00002	0.00001	0.00001	0.00008	0.00001	0.00001	0.00002	0.00000	0.00002
Clostridium argentinense	0.00003	0.00002	0.00001	0.00005	0.00002	0.00002	0.00003	0.00004	0.00001	0.00002	0.00002	0.00002	0.00002	0.00011	0.00002	0.00002	0.00006	0.00001	0.00004
Clostridium autoethanogenum	0.00000	0.00000	0.00000	0.00000	0.00000	0.00001	0.00000	0.00000	0.00000	0.00000	0.00000	0.00000	0.00000	0.00001	0.00000	0.00000	0.00000	0.00000	0.00000
Clostridium baratii	0.00004	0.00002	0.00003	0.00004	0.00002	0.00002	0.00003	0.00004	0.00001	0.00002	0.00002	0.00003	0.00001	0.00016	0.00002	0.00008	0.00003	0.00002	0.00004
Clostridium beijerinckii	0.00003	0.00003	0.00003	0.00006	0.00004	0.00001	0.00004	0.00006	0.00001	0.00003	0.00004	0.00003	0.00001	0.00012	0.00003	0.00007	0.00005	0.00002	0.00007
Clostridium bornimense	0.00005	0.00002	0.00002	0.00007	0.00003	0.00003	0.00004	0.00004	0.00001	0.00003	0.00002	0.00003	0.00001	0.00015	0.00003	0.00003	0.00005	0.00001	0.00004
Clostridium botulinum	0.00013	0.00007	0.00010	0.00019	0.00011	0.00005	0.00009	0.00016	0.00007	0.00007	0.00008	0.00011	0.00006	0.00057	0.00009	0.00019	0.00014	0.00003	0.00018
Clostridium butyricum	0.00009	0.00005	0.00005	0.00013	0.00006	0.00004	0.00010	0.00049	0.00003	0.00007	0.00007	0.00008	0.00003	0.00022	0.00006	0.00007	0.00009	0.00004	0.00011
Clostridium carboxidivorans	0.00002	0.00001	0.00001	0.00003	0.00002	0.00000	0.00003	0.00004	0.00001	0.00001	0.00001	0.00002	0.00001	0.00009	0.00002	0.00002	0.00002	0.00001	0.00003
Clostridium cellulovorans	0.00004	0.00002	0.00002	0.00005	0.00003	0.00001	0.00004	0.00004	0.00001	0.00003	0.00002	0.00003	0.00001	0.00013	0.00003	0.00003	0.00006	0.00002	0.00007
Clostridium chauvoei	0.00001	0.00001	0.00001	0.00001	0.00002	0.00001	0.00001	0.00002	0.00000	0.00001	0.00001	0.00001	0.00001	0.00010	0.00002	0.00002	0.00002	0.00001	0.00001
Clostridium cochlearium	0.00001	0.00001	0.00001	0.00001	0.00001	0.00000	0.00001	0.00001	0.00000	0.00001	0.00001	0.00001	0.00000	0.00008	0.00001	0.00002	0.00001	0.00001	0.00003
Clostridium diolis	0.00000	0.00000	0.00000	0.00000	0.00000	0.00000	0.00000	0.00001	0.00000	0.00000	0.00000	0.00000	0.00000	0.00000	0.00000	0.00001	0.00000	0.00000	0.00000
Clostridium drakei	0.00002	0.00000	0.00001	0.00002	0.00001	0.00001	0.00001	0.00001	0.00000	0.00001	0.00001	0.00001	0.00000	0.00003	0.00001	0.00001	0.00001	0.00001	0.00001
Clostridium estertheticum	0.00003	0.00002	0.00001	0.00006	0.00002	0.00002	0.00003	0.00003	0.00001	0.00002	0.00001	0.00003	0.00000	0.00009	0.00002	0.00004	0.00003	0.00001	0.00004
Clostridium formicaceticum	0.00004	0.00001	0.00001	0.00003	0.00001	0.00001	0.00002	0.00002	0.00001	0.00001	0.00001	0.00001	0.00001	0.00006	0.00002	0.00001	0.00002	0.00000	0.00002
Clostridium intestinale	0.00002	0.00001	0.00001	0.00003	0.00001	0.00001	0.00002	0.00002	0.00001	0.00002	0.00002	0.00002	0.00001	0.00012	0.00002	0.00003	0.00004	0.00001	0.00004
Clostridium isatidis	0.00001	0.00002	0.00001	0.00002	0.00001	0.00001	0.00001	0.00003	0.00001	0.00001	0.00001	0.00001	0.00001	0.00012	0.00002	0.00003	0.00002	0.00000	0.00003
Clostridium kluyveri	0.00004	0.00002	0.00004	0.00004	0.00003	0.00002	0.00003	0.00003	0.00002	0.00002	0.00003	0.00003	0.00002	0.00012	0.00003	0.00005	0.00006	0.00002	0.00006
Clostridium ljungdahlii	0.00000	0.00000	0.00000	0.00000	0.00000	0.00000	0.00000	0.00000	0.00000	0.00000	0.00000	0.00000	0.00000	0.00001	0.00000	0.00000	0.00000	0.00000	0.00000
Clostridium novyi	0.00003	0.00002	0.00002	0.00005	0.00003	0.00001	0.00003	0.00004	0.00001	0.00003	0.00002	0.00003	0.00001	0.00024	0.00003	0.00004	0.00003	0.00001	0.00004
Clostridium pasteurianum	0.00005	0.00004	0.00004	0.00008	0.00005	0.00003	0.00005	0.00008	0.00008	0.00005	0.00004	0.00008	0.00003	0.00022	0.00006	0.00007	0.00007	0.00002	0.00009
Clostridium perfringens	0.00013	0.00006	0.00009	0.00021	0.00011	0.00006	0.00007	0.00026	0.00012	0.00010	0.00018	0.00007	0.00005	0.00045	0.00009	0.00013	0.00012	0.00011	0.00015
Clostridium saccharobutylicum	0.00003	0.00002	0.00002	0.00005	0.00003	0.00003	0.00003	0.00004	0.00002	0.00002	0.00002	0.00001	0.00001	0.00011	0.00003	0.00001	0.00003	0.00000	0.00005
Clostridium saccharoperbutylaceton	0.00005	0.00003	0.00004	0.00008	0.00004	0.00002	0.00004	0.00005	0.00001	0.00003	0.00004	0.00005	0.00001	0.00017	0.00004	0.00006	0.00004	0.00002	0.00006
Clostridium scatologenes	0.00001	0.00001	0.00000	0.00001	0.00001	0.00000	0.00001	0.00001	0.00000	0.00001	0.00001	0.00001	0.00000	0.00003	0.00001	0.00001	0.00001	0.00000	0.00002
Clostridium septicum	0.00001	0.00001	0.00001	0.00003	0.00002	0.00001	0.00001	0.00006	0.00001	0.00001	0.00002	0.00001	0.00001	0.00014	0.00001	0.00004	0.00002	0.00000	0.00002
Clostridium sp. AWRP	0.00002	0.00001	0.00003	0.00003	0.00001	0.00001	0.00002	0.00002	0.00000	0.00002	0.00001	0.00002	0.00001	0.00004	0.00002	0.00004	0.00003	0.00001	0.00003
Clostridium sp. BNL1100	0.00005	0.00003	0.00002	0.00007	0.00004	0.00002	0.00005	0.00005	0.00002	0.00003	0.00003	0.00004	0.00002	0.00011	0.00004	0.00001	0.00007	0.00001	0.00007
Clostridium sp. CT4	0.00002	0.00002	0.00003	0.00004	0.00002	0.00001	0.00004	0.00003	0.00001	0.00003	0.00002	0.00004	0.00001	0.00011	0.00002	0.00002	0.00004	0.00001	0.00004
Clostridium sp. DL-VIII	0.00006	0.00002	0.00003	0.00006	0.00003	0.00003	0.00006	0.00004	0.00001	0.00004	0.00003	0.00005	0.00001	0.00014	0.00003	0.00002	0.00007	0.00002	0.00006
Clostridium sp. JN-1	0.00002	0.00001	0.00002	0.00003	0.00002	0.00000	0.00002	0.00001	0.00001	0.00002	0.00002	0.00001	0.00001	0.00010	0.00001	0.00002	0.00004	0.00001	0.00004
Clostridium sp. JN-9	0.00004	0.00001	0.00002	0.00005	0.00003	0.00001	0.00005	0.00004	0.00001	0.00003	0.00002	0.00003	0.00002	0.00010	0.00002	0.00001	0.00005	0.00001	0.00006
Clostridium sp. JN500901	0.00001	0.00001	0.00001	0.00002	0.00002	0.00000	0.00001	0.00003	0.00001	0.00001	0.00001	0.00001	0.00000	0.00011	0.00002	0.00003	0.00002	0.00000	0.00003
Clostridium sp. MF28	0.00000	0.00000	0.00000	0.00000	0.00000	0.00000	0.00000	0.00000	0.00000	0.00000	0.00000	0.00000	0.00000	0.00000	0.00000	0.00000	0.00000	0.00000	0.00000
Clostridium sp. SYSU GA15002	0.00004	0.00002	0.00002	0.00007	0.00003	0.00002	0.00003	0.00004	0.00001	0.00004	0.00003	0.00003	0.00001	0.00012	0.00003	0.00001	0.00008	0.00001	0.00008
Clostridium taeniosporum	0.00001	0.00001	0.00001	0.00003	0.00002	0.00001	0.00003	0.00003	0.00001	0.00002	0.00001	0.00003	0.00000	0.00009	0.00001	0.00001	0.00003	0.00001	0.00003
Clostridium tetani	0.00003	0.00002	0.00002	0.00003	0.00001	0.00001	0.00003	0.00003	0.00001	0.00002	0.00002	0.00003	0.00000	0.00014	0.00002	0.00006	0.00002	0.00000	0.00003
Clostridium tyrobutyricum	0.00001	0.00002	0.00002	0.00002	0.00002	0.00000	0.00002	0.00002	0.00001	0.00001	0.00001	0.00001	0.00001	0.00008	0.00002	0.00001	0.00003	0.00001	0.00002
Clostridium sporogenes	0.00000	0.00000	0.00000	0.00000	0.00000	0.00000	0.00000	0.00000	0.00000	0.00000	0.00000	0.00000	0.00000	0.00000	0.00000	0.00000	0.00000	0.00000	0.00000
Clostridium;NA	0.00023	0.00013	0.00017	0.00028	0.00022	0.00010	0.00021	0.00032	0.00008	0.00018	0.00015	0.00021	0.00006	0.00114	0.00018	0.00023	0.00029	0.00008	0.00032

	Subject																		
Blautia	s1	s2	s3	s4	s5	s6	s7	s8	s9	s10	s11	s12	s13	s14	s15	s16	s17	s18	s19
**Blautia sp. SC05B48**	**0.0405**	**0.0138**	**0.0181**	**0.0870**	**0.0374**	**0.0144**	**0.0164**	**0.0175**	**0.0022**	**0.0100**	**0.0104**	**0.0111**	**0.0036**	**0.0273**	**0.0092**	**0.0026**	**0.0423**	**0.0112**	**0.0680**
Blautia hansenii	0.0017	0.0011	0.0015	0.0028	0.0022	0.0006	0.0021	0.0031	0.0011	0.0008	0.0019	0.0017	0.0007	0.0025	0.0012	0.0005	0.0024	0.0101	0.0022
Blautia producta	0.0026	0.0009	0.0018	0.0022	0.0027	0.0009	0.0043	0.0033	0.0062	0.0018	0.0039	0.0027	0.0021	0.0026	0.0026	0.0038	0.0023	0.0032	0.0038
Blautia sp. N6H1-15	0.0027	0.0010	0.0011	0.0040	0.0017	0.0007	0.0026	0.0039	0.0011	0.0007	0.0022	0.0020	0.0008	0.0022	0.0016	0.0007	0.0020	0.0013	0.0020
Blautia sp. YL58	0.0000	0.0000	0.0000	0.0000	0.0000	0.0000	0.0000	0.0000	0.0000	0.0000	0.0000	0.0000	0.0000	0.0000	0.0000	0.0000	0.0000	0.0000	0.0000
Blautia;NA	0.0015	0.0008	0.0010	0.0022	0.0014	0.0006	0.0026	0.0020	0.0014	0.0009	0.0018	0.0014	0.0007	0.0022	0.0013	0.0010	0.0020	0.0013	0.0020

	Subject																		
Eggerthella	s1	s2	s3	s4	s5	s6	s7	s8	s9	s10	s11	s12	s13	s14	s15	s16	s17	s18	s19
**Eggerthella lenta**	**0.0007**	**0.0009**	**0.0011**	**0.0028**	**0.0009**	**0.0046**	**0.0020**	**0.0012**	**0.0129**	**0.0027**	**0.0042**	**0.0011**	**0.0002**	**0.0004**	**0.0044**	**0.0039**	**0.0031**	**0.0007**	**0.0010**
Eggerthella sp. YY7918	0.0000	0.0000	0.0000	0.0001	0.0000	0.0000	0.0000	0.0000	0.0000	0.0001	0.0000	0.0000	0.0001	0.0002	0.0001	0.0000	0.0002	0.0000	0.0001
Eggerthella;NA	0.0000	0.0000	0.0000	0.0000	0.0000	0.0000	0.0000	0.0000	0.0000	0.0000	0.0000	0.0000	0.0000	0.0000	0.0000	0.0000	0.0000	0.0000	0.0000

	Subject																		
Barnesiella	s1	s2	s3	s4	s5	s6	s7	s8	s9	s10	s11	s12	s13	s14	s15	s16	s17	s18	s19
**Barnesiella viscericola**	**0.00173**	**0.00061**	**0.00181**	**0.00115**	**0.00062**	**0.00132**	**0.00118**	**0.00049**	**0.00023**	**0.00097**	**0.00069**	**0.00096**	**0.00201**	**0.00198**	**0.00123**	**0.00094**	**0.00066**	**0.00052**	**0.00097**

	Subject																		
Butyricimonas	s1	s2	s3	s4	s5	s6	s7	s8	s9	s10	s11	s12	s13	s14	s15	s16	s17	s18	s19
**Butyricimonas faecalis**	**0.0016**	**0.0041**	**0.0012**	**0.0029**	**0.0018**	**0.0056**	**0.0007**	**0.0020**	**0.0006**	**0.0034**	**0.0049**	**0.0043**	**0.0062**	**0.0076**	**0.0013**	**0.0041**	**0.0027**	**0.0012**	**0.0012**

	Subject																		
Collinsella	s1	s2	s3	s4	s5	s6	s7	s8	s9	s10	s11	s12	s13	s14	s15	s16	s17	s18	s19
**Collinsella aerofaciens**	**0.04814**	**0.02807**	**0.01038**	**0.02709**	**0.01433**	**0.03724**	**0.05531**	**0.0018**	**3.2E-05**	**0.00023**	**8E-05**	**0.00045**	**0.00011**	**0.04884**	**0.00088**	**0.00029**	**0.02341**	**0.00023**	**0.00144**

	Subject																		
Faecalibacterium	s1	s2	s3	s4	s5	s6	s7	s8	s9	s10	s11	s12	s13	s14	s15	s16	s17	s18	s19
**Faecalibacterium prausnitzii**	**0.0751**	**0.0288**	**0.0832**	**0.0585**	**0.0641**	**0.0253**	**0.0773**	**0.0960**	**0.0110**	**0.0279**	**0.0026**	**0.0870**	**0.0256**	**0.1414**	**0.0799**	**0.0057**	**0.0189**	**0.0451**	**0.0986**

	Subject																		
Odoriobacter	s1	s2	s3	s4	s5	s6	s7	s8	s9	s10	s11	s12	s13	s14	s15	s16	s17	s18	s19
**Odoribacter splanchnicus**	**0.0054**	**0.0054**	**0.0058**	**0.0043**	**0.0055**	**0.0070**	**0.0090**	**0.0048**	**0.0002**	**0.0046**	**0.0027**	**0.0113**	**0.0124**	**0.0082**	**0.0048**	**0.0036**	**0.0061**	**0.0020**	**0.0049**

Supplementary Figure 3. Intestinal microbiome identification by shotgun metagenomic analysis (Study 2)

Dark green indicates greater abundance. When multiple bacterial strains were detected within a single genus, the strain with the highest abundance among all participants was selected for individual regression analysis.
